# Anti-herpes virus activity of *lactobacillus’* postbiotics

**DOI:** 10.37796/2211-8039.1277

**Published:** 2022-03-01

**Authors:** Neli Vilhelmova-Ilieva, Georgi Atanasov, Lora Simeonova, Lili Dobreva, Kapka Mancheva, Madlena Trepechova, Svetla Danova

**Affiliations:** a The Stephan Angeloff Institute of Microbiology – Bulgarian Academy of Sciences, Member of the Institut Pasteur International Network, 26, Georgi Bonchev Str., 1113 Sofia, Bulgaria; b Institute of Biodiversity and Ecosystem Research – Bulgarian Academy of Sciences, 25, Georgi Bonchev, Str., 1113 Sofia, Bulgaria; c Institute of Biophysics and Biomedical Engineering, Bulgarian Academy of Sciences, 23, Georgi Bonchev, Str., 1113 Sofia, Bulgaria

**Keywords:** Antiviral activity, Herpes simplex virus type 1, *Lactobacillus* probiotics, Postbiotics

## Abstract

**Background:**

Recently various lactic acid bacteria (LAB) and their post-metabolites have shown many positive effects on human and animal welfare. They appear to be beneficial in different disorders and pathological conditions, including in a broad-spectrum of infectious diseases.

**Aim:**

To estimate *in vitro* the anti-herpes simplex activity of 11 postbiotic samples (lysates or cell-free supernatants - CFS) produced during the fermentation of six candidate-probiotic *Lactobacillus* strains isolated from Bulgarian fermented milk products.

**Materials and methods:**

*In vitro* protocols for assessment of different LAB samples on the Herpes simplex virus type 1 (HSV-1) replication, adsorption and virucidal effects were applied using MDBK cells.

**Results:**

Four of the studied LAB samples expressed a statistically significant inhibition of the replication of HSV-1. The highest selective index (79.75) was calculated for the post-metabolites of *Lactiplantibacillus plantarum*, followed by a high molecular fraction of cell-derived fragments of *Limosilactobacillus fermentum* culture (S6) (SI = 34.63), CFS from late exponential *L. plantarum* (SI = 28.26) and neutralized CFS from *L. fermentum* (SI = 28.11). Pronounced virucidal activities of the postbiotics S1, S11 (*L. fermentum*), S3 (*L. plantarum*) and S6 (*L. fermentum*) were recorded, too. The inhibitory effect of the majority of the samples on the stage of adsorption of the virus to MDBK cells was remarkable. In addition, almost all of the postbiotics exerted a protective effect on healthy cells and significantly reduced viral yield at subsequent infection.

**Conclusion:**

Pre-selected *Lactobacillus* strains demonstrated strain-specific effects against HSV-1. These postbiotics influence different stages of viral infection in cell cultures and their promising characteristics are currently evaluated.

## 1. Introduction

Herpes simplex virus type 1 (HSV-1) infection is a widespread viral disease in the human population with more than 90% of the individuals being seropositive to the pathogen [1,2]. The virus is transmitted via close contact with a carrier, whether the person be asymptomatic or with the typical manifestation. After a primary infection acquired usually in the early childhood, the causative agent is translocated through the peripheral sensory nerves to the cranial nerve ganglion, where it establishes a lifelong latent persistence [3]. When exposed to different types of stimulation (temperature variation, prolonged exposure to sunlight, psychological stress, physical overload, or other active contagious diseases in the body, etc.), the latent virus can be reactivated and cause a recurrent disease. Herpes simplex virus (HSV) type 1 causes mainly symptoms affecting the mouth, throat, lips, eyes and the central nervous system but can also affect various areas of the skin. Recently incidences of genital herpes caused by HSV-1, as a result of transmission through oral contact in this area, have been reported [4–6]. Severe herpes-related conditions include encephalitis, meningitis, neonatal herpes [7,8], as well as disseminated infections in immunocompromised patients have been described [9–11].

The specific anti-herpes chemotherapy relies on nucleoside analogues which shorten the duration of symptoms and lead to faster healing of lesions. Currently, the most widely used drug is acyclovir (ACV), which is also the first selective inhibitor of HSV developed [12,13]. A disadvantage of these antivirals, both having been used as a first line and continuously applied medication in acute and chronic cases, respectively, is the relatively rapid formation of resistant mutants in patients with a compromised immune system who are particularly at risk. This naturally leads to the necessity to increase the therapeutic doses but unfortunately such a treatment quite often inevitably fails [14–17]. Therefore, new therapeutics, other than those targeted by nucleoside analogues, are needed to be enacted at different stages of the viral replication. Products of natural origin are being considered as possible candidates since they are better absorbed and tolerated by cells and their therapeutic doses are relatively low. Over the last few decades special attention has been paid to probiotics and their postmetabolites isolated from various dairy products [18]. Many of their biological activities, such as antimicrobial, antifungal, anti-tumor, immunomodulatory and still many others, have been proven [19]. Some probiotic postbiotics (the active metabolites produced during the fermentation) and/or parabiotics (cell-derived fragments) have shown antiviral activity. The postbiotics/parabiotics produced from live bacteria or released into environment after probiotic lysis may bring beneficial effects to the host [20]. These are presented by a wide range of effector molecules such as short chain fatty acids (SCFAs), bacteriocins, small molecules - proteins/peptides, neurotransmitters, etc. [21]. Bacteriocins produced by *Lactobacillus delbrueckii* subsp. *bulgaricus* specifically inhibit the replication of influenza virus A/chicken/Germany, strain Weybridge (H_7_N_7_) and strain Rostock (H_7_N_1_) [22]. A significant decrease in influenza H_1_N_1_ virus titers has also been observed in the treatment with *Lactobacillus plantarum* L-137 of infected mice, inducing a pro-inflammatory response [23]. When administering *Lactobacillus rhamnosus* GG decreased the incidence of respiratory viral infections [24] and a reduction in influenza-like symptoms was recorded with *Lactobacillus acidophilus* strain NCFM [25]. Consistent and broad *in vitro* anti-HIV activity has been reported for the carbocyclic lantibiotic Labyrinthopeptin A1 [26]. Glycerol monolaurate, a factor analogously secreted by *Lactobacillus*, has virucidal activity against enveloped viruses, including HIV-1 [27]. Inhibitory activity against murine norovirus (MNV) has also been investigated using a mixture of metabolites of *Lactilactobacillus curvatus* 1 [28]. Exopolysaccharides produced by lactic acid bacteria from genera *Pediococcus*, *Leuconostoc* and *Lactobacillus* show antiviral activity versus human Adenovirus type 5 [29]. Strains of *L*. *plantarum* and *Lactobacillus amylovorus* exhibit good inhibitory effects against Echovirus 7 (E7) and E19 [30]. *Bifidobacterium longum* and *L. plantarum* in combination with *Chlorella soroquiniana* in dairy products have exerted anti-rotavirus activity [31]. However, limited data exists on the mode of anti-Herpes simplex virus action of *Lactobacillus*-derived post-metabolites/parabiotics. The lack of therapy capable of eliminating herpes infections as well as the generation of resistant mutants in the current treatment approach requires the development of new agents with a mode of action alternative to nucleoside analogues. Having in mind the above stated objectives, 11 postbiotic samples obtained from 6 *Lactobacillus* strains with probiotic properties were investigated as anti-Herpes simplex virus type 1 agents at different stages of replication.

## 2. Materials and methods

### 2.1. Lactobacilli and post-metabolites tested

Lactic acid bacteria (LAB) in the present study are part of a laboratory collection of The Stephan Angeloff Institute of Microbiology, Bulgaria. They were pre-selected on the basis of previously estimated broad spectrum of anti-bacterial activity [32,33]. All strains were stored at −20°C in MRS broth, supplemented with glycerol (20% v/v). Prior to the assay, all lactobacilli were pre-cultured twice in MRS broth (Merck, Germany) at 37°C for 24–48 h.

A total of 11 samples (named S1–S11) (Table 1) derived from 6 *Lactobacillus* strains and a multi-bacterial formula of 8 LAB strains were assessed for anti-HSV activity. Two vaginal strains *Limosilactobacillus fermentum (L.f*. S3) and *Ligilactobacillus salivarius (L.s. 1)*, four *Lactiplantibacillus plantarum* (*L.pl.)* strains isolated from Bulgarian fermented milk products such as home-made sample of a yogurt-like product “katak” [34] – *L.pl*. L3 and L14 and from artisanal green cheese (produced only in the village Tcherni vit, Bulgaria) – strains *L. pl* 3 ZS and 4 ZS [33].

Four of the tested samples were filtered acid cell-free supernatants (aCFS) from exponential (24 h) cultures of the strains *L. pl*. L3 (sample S1); *L. pl*. L14 (S2), *L. pl*. ZS3 (S3) and *L. pl*. ZS4 (S4) in MRS broth (Merck, Germany). The other samples were different fractions from LAB postmetabolites in the same laboratory medium as follows: the sample S5 - a low molecular mass fraction <30,000 Da, obtained with centrifugation (Hermle, Germany at 5000 rpm, 5 min) of a CFS of *L. fermentum* S3 culture, using Amicone centrifuge tube, with cut-off membrane 30,000 Da; the sample S6 – a high molecular mass fraction >30,000 Da, obtained as the S5 sample from *L. fermentum* S3; sample S7 - the supernatant obtained by centrifugation at 3000×*g* from thermally destroyed (15 min at 110 °C) exponential co-culture - Mix 8 (containing 8 *Lactobacillus* strain co-cultivated 24 h in MRS broth); the sample S8 – a supernatant, obtained by centrifugation at 3000×*g* from ultra-sonicated *L. salivarius* 1 culture, cultivated 24 h in MRS broth at 37 °C; S9 – neutralized with 5 M NaOH cell-free supernatants (pH 6.0) from a 24 h - culture of *L. fermentum* S3- in MRS broth (pH 6.5); the sample S10 - a supernatant, obtained by centrifugation at 3000×*g* from ultra-sonicated *L. fermentum S3* culture, cultivated 24 h in MRS broth at 37 °C and the sample S11 – a CFS from 24 h *L. fermentum* S3 culture in MRS broth. All samples were filter sterilized (0.22 μm) and stored at −20 °C prior to the experiments.

The Bradford reagent (Sigma) with Coomassie dye-binding assays was used for protein quantification (mg/ml) of all samples, according to instructions of the manufacturer.

### 2.2. Virus and cells

Madin–Darby bovine kidney (MDBK) cells were obtained from National Bank for Industrial Microorganisms and Cell Cultures, Sofia, Bulgaria. The cell lines were grown in DMEM medium containing 10% v/v fetal bovine serum (Gibco BRL, USA), supplemented with 10 mM HEPES buffer (Merck, Germany) and antibiotics (penicillin 100 IU/ml, streptomycin 100 μg/ml) in CO_2_ incubator (HERA cell 150, Heraeus, Germany) at 37 °C/5% CO_2_.

Herpes simplex virus type 1, Victoria strain (HSV- 1) was received from Prof. S. Dundarov, National Center of Infectious and Parasitic Diseases, Sofia. Virus was replicated in monolayer MDBK cells in a maintenance solution DMEM Gibco BRL, Paisley, Scotland, UK, plus 0.5% fetal bovine serum Gibco BRL, Scotland, UK. Infectious titer of stock virus was 10^7.5^CCID_50_/ml.

### 2.3. Cytotoxicity of LAB samples

Confluent monolayer cell culture in a 96-well plates (Costar®, Corning Inc., Kennebunk, ME, USA) was treated with 0.1 ml/well-containing a maintenance medium containing no (untreated control)/or decreasing concentrations of the tested products. Cells were incubated at 37 °C and 5% CO_2_ for 48 h. After microscopic evaluation, the medium containing the test compound was removed; cells were washed stained with neutral red and then incubated at 37 °C for 3 h. After the incubation, the neutral red dye was removed; the cells were washed with PBS, and 0.15 ml/well desorb solution (1% glacial acetic acid and 49% ethanol in distilled water) was added. The optical density (OD) of each well was read at 540 nm in a microplate reader (Biotek Organon, West Chester, PA, USA). The 50% cytotoxic concentration (CC_50_) was defined as the material concentration that reduced the cell viability by 50% when compared to untreated control. Each sample was tested in triplicate with four cell culture wells per test sample.

The maximum tolerable concentration (MTC) of the products, which is the concentration at which they do not affect the cell monolayer and it looks like the cells in the control (untreated with extract) was also determined.

### 2.4. Antiviral activity

Cytopathic effect (CPE) inhibition test was used for assessment of antiviral activity of the extracts. Confluent cell monolayer in 96-well plates infected with 100-cell culture infectious dose 50% (CCID_50_) in 0.1 ml (HSV-1). After 60 min of virus adsorption, each of the products was added in various concentrations and cells were incubated for 48 h at 37 °C. The cytopathic effect was determined using a neutral red uptake assay and the percentage of CPE inhibition for each concentration of the test sample was calculated using the following formula:


$$\%\;\text{CPE}=[{\text{OD}}_{\text{test sample}}-{\text{OD}}_{\text{virus control}}]/[{\text{OD}}_{\text{toxicity control}}-{\text{OD}}_{\text{virus control}}]\times 100$$

where OD_test sample_ is the mean value of the ODs of the wells inoculated with virus and treated with the test sample in the respective concentration, OD_virus control_ is the mean value of the ODs of the virus control wells (with no compound in the medium) and OD_toxicity control_ is the mean value of the ODs of the wells not inoculated with virus but treated with the corresponding concentration of the test sample. The 50% inhibitory concentration (IC_50_) was defined as the concentration of the material that inhibited 50% of viral replication when compared to the virus control. The selectivity index (SI) was calculated from the ratio CC_50_/IC_50._

### 2.5. Virucidal assay

Contact samples of 1 ml containing HSV-1 (10^4^ CCID_50_), as well as tested compound in its maximum tolerable concentration in a 1:1 ratio was stored at room temperature for different time intervals (15, 30, 60, 90 and 120 min). Then, the residual infectious virus content in each sample was determined by the end-point dilution method and Δlgs as compared to the untreated controls were evaluated.

### 2.6. Virus attachment

Twenty four well cell culture plates containing monolayer of MDBK cells were pre-chilled at 4 °C and were inoculated with 10^4^ CCID_50_ of HSV-1 for adsorption at 4 °C and treated in parallel with MTC of the extract. At various intervals (15, 30, 45 and 60 min) cells were washed with PBS in order to remove both the compound and the unattached virus, then overlaid with maintenance medium and incubated at 37 °C for 24 h. Following triple freezing and thawing the infectious virus, titer of each sample was determined by the end-point dilution method. Each sample was prepared in triplicate.

### 2.7. Pre-treatment of MDBK cells

Monolayers of MDBK cells pre-grown into 24-well cell culture plates (CELLSTAR, Greiner Bio-One) (2 × 10^6^ cells per well) were treated for 15, 30, 60, 90 and 120 min at concentration of MTC of the extract in the maintenance medium (1 ml per well). The extract was then removed and the cells were washed with phosphate-buffered saline (PBS) and inoculated with HSV-1 (1000 CCID_50_ in 1 ml per well). After 60 min of absorption, the non-absorbed virus was removed and the cells were covered with maintenance medium. The culture plates were incubated at 37 °C for 24 h and, after triple freezing and thawing, the infectious viral titers were determined by the end-point dilution method. Δlgs were evaluated compared to viral control (untreated with compounds).

### 2.8. Statistical analyses

Determination of standard deviation (SD) was done using the program Origin Pro 7.5. Student’s t-test compared two groups of results. The final data sets were analyzed with the Graph Pad Prism 4 program.

## 3. Results

The cytotoxicity assessment of the eleven LAB samples (post-metabolites and cell-derived fragments) from 6 pre-selected *Lactobacillus* strains (Table 1) was performed to exclude any possible overlapping cytopathic effect of the samples and changes induced purely by the virus itself. The assay was performed on MDBK monolayer cell line (Table 2).

One of the tested LAB (sample S8, which is derived from 8 active strains) showed cytotoxicity comparable to ACV and five LABs (samples S1, S5, S6, S9 and S11) exhibited about two-fold higher toxicity than the reference nucleoside analogue. Four of the tested samples (S2, S3, S4 and S7) were about 20 times more toxic than ACV and only one of the products (S10) showed CC_50_ values which is about 3 times higher than that of reference substance.

In order to discard a probable effect of cytotoxicity of the MRS medium used for LAB cultivation, we also assessed its influence on the monolayer of MDBK cells. The results showed no toxicity, even of the stock solution (Table 2). The maximum tolerated concentration of substances which does not induce visible changes in the cell monolayer was also determined.

The effect of LAB post-metabolites on viral replication in MDBK cells was evaluated, too (Table 2). Four of the tested samples possessed remarkable anti-herpes virus activity. The highest selective index (79.75) and therefore the most pronounced effect on the internal replicative cycle of HSV-1 was determined for aCFS of *L. plantarum* L3 (named S1 sample). Although being established as the most potent among all the tested post-metabolites, its effect was 10 times weaker than that of ACV. Next in activity was sample S6 with a selective index of 34.63, followed by the aCFS of *L. plantarum* L4ZS (S4 sample) and neutralized CFS of *L. fermentum* S3 (named S9) with almost identical selective indices, 28.26 and 28.11, respectively. These results clearly showed that an active metabolite different from lactic acid is probably produced during the fermentation. We observed weak anti-viral effect for S11 (SI = 10.49) and insignificant activities for S2 (SI = 3.51), S3 (SI = 2.15) and S10 (SI = 1.5). Three of the tested samples −S5, S7 and S8 did not show any effect on the viral replicative cycle of HSV-1 (Table 2).

The pronounced activity of some of the post-metabolites against the intracellular replicative cycle of HSV-1 inspired us to analyze whether they have an effect on the extracellular virions of HSV-1. The results in Table 3 indicate that at 15 min of virus exposure none of the products shows any activity. At 30 min exposure only S1 and S3 exert some moderate activity, and S3 at 60 min exposure showed Δlg = 1.75, which is sustained until 120th min (the last studied time interval) as compared to virus control. Weak activity at 60 min was determined for S6, S7 and S9 with S7 and S9 showing no greater virucidal properties up to 120th min, while S6 at 120 min showed an activity of Δlg = 1.75. The strongest virucidal effect of all examined products was recorded for S1 and S11 at 120 min with Δlg = 2 compared to vehicle-treated group.

The effect of post-metabolites on the attachment of HSV-1 virions to susceptible MDBK cells is summarized in Table 4. At 15 min of exposure none of the products showed any effect on the stage of adsorption of the virus on the cell membrane. At the next time interval of 30 min S1 exerted remarkable activity of Δlg = 4.75. Other products showing virucidal activity at this time interval were S9 (Δlg = 2.0), S6 (Δlg = 1.75) and S11 (Δlg = 1.75). After 45 min of contact with the substances the most impressive effect was calculated for S1 (Δlg = 4.75), S6, S8 and S11 (Δlg = 3.0). S7 (Δlg = 2.5) and S9 (Δlg = 2.0). S2, S3 (Δlg = 1.5) and S4, S10 (Δlg = 1.25) had low activity. At 60 min from the effect of the post-metabolites a statistically strong inhibitory effect on the adsorption stage of HSV-1 virions to sensitive MDBK cells was found for all tested products except for S5 and S4.

The most significant effect was demonstrated by S6, S11 (Δlg = 5.5) and S1, S7, S8 (Δlg = 5.0). The influence of S2, S9 (Δlg = 2.0) and S3, S10 (Δlg = 1.75) was also estimated as strong.

The protective effect of post-metabolites established on the stage of adsorption of viral particles to host cells, brought us to the idea to test the samples’ behavior on uninfected MDBK cells when applied prior to viral infection (Table 5). As early as 15 min of incubation of the LAB samples with uninfected cells the S9 (Δlg = 2.5), S7 and S10 (Δlg = 1.75) significantly preserved the cells from subsequent herpes infections. At 30 min of treatment with probiotic post-metabolites all of them, except S5, exerted defensive effects to the cells. They varied for the individual products: S9 (Δlg = 2.5); S2, S11 (Δlg = 2.25); S6 (Δlg = 2.0); S1, S3, S8, S10 (Δlg = 1.75) and a moderate effect was expressed by S4, S7 (Δlg = 1.5). It was enhanced by increasing the exposure time and a clear protection was observed after 120 min of treatment with the products (except for S5, which did not show activity in any of the studied time intervals). The most significant were the protective properties shown by acid CFS from *L. plantarum* strains in MRS - S1, S4 (Δlg = 3.5); followed by a combination of 8 pre-selected candidate probiotic strains - S8 (Δlg = 3.25) and S2, S6, S11 (Δlg = 3.0). Values for S3, S7, S10 samples were evaluated as Δlg = 2.0 (Table 5).

## 4. Discussion

One of the milestone challenges in developing antiviral chemotherapeutic agents comes from the natural conjunction between the viral replicative mechanisms and the regular host cell processes. Thus, targets for effective antiviral drugs are those virus-specific structures and functions that determine viral replication but are not essential for the cells and host organism’s normal activities.

The first stage that can be affected by any antiviral candidate is the time before the pathogen enters the cell, i.e. the studied samples act on extracellular virions. The reduction in the ability of virions to infect sensitive cells is based on the capacity of particular compounds to inactivate extracellular viral particles nonspecifically by denaturating their proteins and/or causing structural changes in supercapsid lipids. Various mechanisms of action of probiotics in viral infections exist but are not fully characterized. Possibly the virucidal effect of the samples S1, S2, S3 and S4 reported in Table 3 is mainly due to the detrimental effect of lactic acid produced during the fermentation in the aCFS we studied. A similar effect has been described by the teams of Tuyama et al., 2006 [35] and Conti et al., 2009 [36] against human simplex virus type 2 (HSV -2). The presence of such a non-specific interaction is also suggested by the lack of a virucidal effect upon exposure to neutralized CFS (in which lactic acid was eliminated with NaOH). The probiotics may combat with pathogens through different barrier mechanisms. LABs appear to be a rich source of biologically active products with diverse structure and biological functions. This research suggests that the post-metabolites produced by selected *Lactobacillu*s strains during the fermentation in MRS broth are able to inhibit HSV-1 infection of MDBK cells at different stages of the replicative cycle. Thus, different samples from acid and neutralized cell-free supernatants of exponential cultures in MRS broth and cells fragments have been estimated. Our hypothesis was that LAB are able to produce different active metabolites during the fermentation, able probably strain-specifically to inhibit virus replication/attachment or infection. Moreover, it has been shown that selected vaginal strains of *Lactobacillus* (*Levilactobacillus brevis* CD2, *L. salivarius* FV2, *L. plantarum* FV9) as well as their cell-free supernatants affect various steps of herpes simplex virus type 2 (HSV-2) replication *in vitro* [36]. Independent studies on antiviral properties of multiple probiotics and their derivatives on the replication of herpes simplex viruses have been conducted [37]. It has been shown that selected vaginal strains of *Lactobacillus*, as well as their cell-free supernatants affect various steps of herpes simplex virus type 2 (HSV-2) replication *in vitro* [36]. The bacterial extract of *Lactobacillus brevis* and fragments of its cell wall has demonstrated a dose-dependent inhibitory effect on HSV-2 proliferation [38].

Next, encouraged by the above-mentioned results, our questioning whether the probiotic samples can induce insusceptibility to viral infection by pre-treatment of uninfected cells found its answer. The obvious protective effect of the tested samples on intact cells observed could be explained by their bacteriocin content. Bacteriocin-like inhibitory substances (BLIS) can block the receptors on the host cell responsible for virus recognition and binding [39]. The defensive mechanism of pre-treatment of cells with metabolic products of the probiotic *L. plantarum* strain N4 against transmissible gastroenteritis Coronavirus has also been proven. The analyzed composition of metabolic products by GC–MS revealed that the major components were sugars [40]. Perhaps the content and action of similar constituents in the LAB samples we studied explain the remarkable prophylactic antiviral properties on healthy cells of almost all tested samples, especially S1, S2, S4, S6, S8, S9 and S11. They reduce subsequent viral infection with a decrease in viral titer by Δlg ≥3. Moreover, the effect was also significant, after the elimination of protective role of lactic acid produced, as it was shown for sample S9 containing nCFS. Concomitantly variants of produced post-metabolites (samples S5, S6, S9 and S11) and parabiotics (S10) derived from the pre-selected *L. fermentum* 3 S strain, were assessed for anti-HSV effects *in vitro*.

Mousavi et al. (2018) assume that the inhibition of the HSV-2 entry into cells occurs because viral particles are trapped by *Lactobacillus crispatus* [41]. The same team suggested the formation of *L. crispatus* protective biofilm on the cell surface that block HSV-2 receptors and prevent the virus from entering cells in the early stages of infection.

Intriguingly, our additional experiments with LAB post-metabolites found potential of samples in terms of inhibition of virus attachment and its subsequent entry and further infection-related events (Table 4).

It has been suggested that bacteriocins secreted by different strains of lactobacilli as positively charged compounds can be adsorbed on the viral capsid by electrostatic interaction, thereby inhibiting viral adsorption to the host cell [42]. The result of such an interaction may be the inhibition of viral adsorption reported by us, as well as the viral effect of some of the samples. The L. *fermentum* S3 strain was preliminarily evaluated as a putative BLIS-producing strain active against Gram (+) and Gram (−) bacteria (unpublished data). Therefore, we prepared different samples to visualize the activity of the obtained postbiotics - low molecular weight samples - S5 or >30,000 Da – S6 and cells-free supernatants of spent cultures -S9 and S11). Thermal destructed cells from exponential culture of *L. salivarius* 1 S – the sample S8, shows a complete absence of antiviral activity, despite the previously proven bacteriocinogenic anti-*Salmonella* activity [33]. Probably the active proteinaceous compound produced by *L. salivarius* 1 S was completely destroyed by high temperature (120 °C).

Herpes viruses induce the expression of a large number of virus-specific enzymes in the infected cells. They mimic the action of functionally related cellular enzymes but differ in a number of biochemical characteristics such as molecular weight, dependence on certain cations, sensitivity to inhibitors, substrate specificity and many others. The most effective therapeutics are those that block the intracellular replicative cycle of the virus by attacking specific viral structures in the infected cell that are necessary for its reproduction.

In this study, the highest selective indices were calculated for samples S1, S4, S6, S9 and S11, which could indicate interruption in one or more important steps of the intracellular replication cycle of HSV-1. This effect is not likely solely as a result of the lactic acid content present in the samples. Sample S9 was also active lacking lactic acid by neutralization in CFS of the exponential culture of one candidate probiotic strain Lf. S3 (Table 2). Serkedzhieva et al. (2000) found that bacteriocin B1 from *Lactobacillus delbrueckii* inhibited certain stages of the intracellular replicative cycle of the virus [22]. Wachsman et al. (2003) show that inhibition of HSV replication by enterocin CRL35 occurs in the late stages of virus replication by preventing the synthesis of gamma protein-glycoprotein D (gD), a structural component of the HSV envelope that is essential for entry of the virus in the host cell [39]. In other studies, a non-protein component contained in the cell wall of *Lactobacillus brevis* also has the ability to reduce HSV-2 replication [38]. Our assays in accordance with other teams, prove an inhibitory effect on viral particles and viral replication of LAB and their postmetabolites. However, the experiments conducted so far represent only the initial stage of this research. It is necessary further to establish in more details the exact chemical composition of postmetabolites, as well as the effect that each ingredient has on viral replicative cycle. Additional studies should be performed to determine the precise mechanism explaining the antiherpes effect observed in the studies.

## 5. Conclusion

All in all, tested *Lactobacillus* strains demonstrated a strain-specific effect against HSV-1. This study assesed the capacity of LABs postmetabolites/cell-derived fragments to influence different stages of viral infection in cell cultures. Our *in vitro* achievements showed new aspects for widespread use of LABs and their postbiotics in the treatment of herpes infections. They could successfully shorten and limit existing herpes recurrence, disrupting both stages of virus replication and restricting the movement and passage of the virus from cell to cell. The protective effect they have on healthy cells is also extremely important protecting them to a significant extent from the entry of viral particles into them. These activities would make LABs and their post metabolites reliable future therapies for herpes infections. Despite the reported decrease in viral yield at different stages of viral cycle, the exact mechanism by which probiotics and their post-metabolites act has not been established so far. This opens a broad perspective in the development and application of probiotics as antiviral agents with a variety of additional biological activities and benefits for human health.

## Figures and Tables

**Table 1:** LAB samples tested in the present work.

Samples	Tested strains	Description of the samples
S1	*L. plantarum* L*3*	Acid CFS from exponential (24 h) cultures in MRS broth (Merck, Germany) at 37 °C.
S2	*L. plantarum* L*14*	
S3	*L. plantarum* L*3ZS*	
S4	*L. plantarum* L*4ZS*	
S5	*L. fermentum S3*	A low molecular mass fraction <30,000 Da obtained with centrifugation (Hermle, Germany at 5000 rpm, 5 min) of aCFS from *L. fermentum* S3 culture in MRS, using Amicone centrifuge tube, with cut-off membrane 30,000 Da.
S6	*L. fermentum S3*	A high molecular mass fraction >30,000 Da obtained with centrifugation (Hermle, Germany at 5000 rpm, 5 min) of aCFS from *L. fermentum* S3 culture in MRS, using Amicone centrifuge tube, with cut-off membrane 30,000 Da.
S7	Mixed LAB culture - Mix 8	A supernatant obtained by centrifugation at 3000×*g* from thermally destroyed (15 min at 110 °C) exponential mixed culture - Mix 8 (containing 8 *Lactobacillus* strains co-cultivated 24 h in MRS broth).
S8	*L. salivarius 1S*	A supernatant, obtained by centrifugation at 3000×*g* from ultra-sonicated *L. salivarius* culture, cultivated 24 h in MRS broth at 37 °C;
S9	*L. fermentum* S3	A neutralized cell-free supernatants (pH 6.0), with 5 M NaOH from a 24 h - culture of *L. fermentum* S3 in MRS broth (pH 6.5);
S10	*L. fermentum* S3	A supernatant, obtained by centrifugation at 3000×*g* from ultra-sonicated *L. fermentum S3* culture, cultivated 24 h in MRS broth at 37 °C
S11	*L. fermentum* S3	Filtered aCFS from exponential (24 h) cultures in MRS broth (Merck, Germany) at 37 °C

**Table 2:** In vitro assessment of cytotoxicity and antiviral activity of LAB post-metabolites.

Tested samples	Cytotoxicity (μg/ml)		Antiviral activity	
	
	CC_50_	MTC	IC_50_ (μg/ml)	SI
S1	131.7 ± 5.34***	2.1	0.8 ± 0.04***	79.75
S2	12.6 ± 1.54***	0.6	1.85 ± 0.06***	3.51
S3	13.3 ± 0.54***	2.0	3.07 ± 0.21***	2.15
S4	13.1 ± 1.85***	0.6	0.23 ± 0.009**	28.26
S5	118.7 ± 3.98***	6.6	–	–
S6	128.0 ± 3.43***	1.8	2.05 ± 0.13***	34.63
S7	253.4 ± 8.21**	12.3	–	–
S8	12.8 ± 2.12***	0.6	–	–
S9	115.8 ± 4.24***	1.9	1.0 ± 0.05***	28.11
S10	849.1 ± 5.32***	120.0	10.0 ± 0.44***	1.5
S11	127.5 ± 6.64***	6.3	6.22 ± 0.52***	10.49
MRS broth	–	157.1	–	–
ACV	291.0 ± 9.4	–	0.33 ± 0.03	881.8

** p<0.001, when comparing the value of each post-metabolite with ACV; Student’s *t*-test.

*** p<0.0001, when comparing the value of each post-metabolite with ACV; Student’s *t*-test.

**Table 3:** Virucidal activity against HSV-1 various of LAB post-metabolites/parabiotics.

Tested samples	Δlg				

	15 min	30 min	60 min	90 min	120 min
S1	0.75	1.0	1.0	1.5	2.0
S2	0	0	1.0	1.0	1.0
S3	0.5	1.0	1.75	1.75	1.75
S4	0.25	0.25	1.0	1.0	1.5
S5	0	0	0.25	0.25	0.25
S6	0.25	0.5	1.5	1.5	1.75
S7	0.25	0.5	1.0	1.0	1.0
S8	0	0.5	1.25	1.25	1.5
S9	0.75	0.75	1.5	1.5	1.5
S10	0.25	0.25	0.25	0.25	0.25
S11	0.25	0.5	0.5	1.0	2.0

**Table 4:** Effect of LAB post-metabolites on viral adsorption of HSV-1 on MDBK cells.

Tested samples	Δlg			

	15 min	30 min	45 min	60 min
S1	1.25	4.75	4.75	5.0
S2	0.25	1.5	1.5	2.0
S3	0.25	1.5	1.5	1.75
S4	0	1.25	1.25	1.25
S5	0	0.25	0.25	0.5
S6	0	1.75	3.0	5.5
S7	0	1.25	3.0	5.0
S8	0	0.5	2.5	5.0
S9	0.25	2.0	2.0	2.0
S10	0.25	0.5	1.25	1.75
S11	0	1.75	3.0	5.5

**Table 5:** Pretreatment of MDBK cells with LAB derived fragments or postmetabolites before HSV-1 infection.

Tested samples	Δlg				

	15 min	30 min	60 min	90 min	120 min
S1	1.0	1.75	2.0	2.5	3.5
S2	0.5	2.25	2.25	2.5	3.0
S3	0.25	1.75	1.75	2.0	2.0
S4	0.25	1.5	2.0	2.25	3.5
S5	0.25	0.25	0.25	0.5	0.5
S6	0.5	2.0	2.25	2.75	3.5
S7	1.5	1.5	1.5	2.0	2.0
S8	1.75	1.75	2.0	2.75	3.25
S9	2.5	2.5	2.5	2.5	3.0
S10	1.75	1.75	1.75	2.0	2.0
S11	0.5	2.25	2.25	2.5	3.0
